# A cross sectional study of HPV type prevalence according to age and cytology

**DOI:** 10.1186/1471-2334-13-53

**Published:** 2013-01-30

**Authors:** Elena Argyri, Stefanos Papaspyridakos, Elpida Tsimplaki, Lina Michala, Evangelia Myriokefalitaki, Issidora Papassideri, Dimitra Daskalopoulou, Ioanna Tsiaoussi, George Magiakos, Efstathia Panotopoulou

**Affiliations:** 1 Department of Virology, “G. Papanikolaou” Research Center of Oncology and Experimental Surgery, Regional Anticancer Oncology Hospital of Athens “St. Savvas”, 171 Alexandras Avenue, 11522, Athens, Greece; 2 1st Department of Obstetrics and Gynaecology, University of Athens, ‘Alexandra’ Hospital, Alexandra, Greece; 3 Department of Cell Biology and Biophysics, Faculty of Biology, University of Athens, Panepistimiopolis, 15784, Athens, Greece; 4 Department of Cytopathology, Regional Anticancer Oncology Hospital of Athens "St. Savvas", 171 Alexandras Avenue, 11522, Athens, Greece; 5 1st Department of Gynaecology, Regional Anticancer Oncology Hospital of Athens "St. Savvas", 171 Alexandras Avenue, 11522, Athens, Greece

**Keywords:** Human papillomavirus (HPV), Prevalence, Typing, Age-distribution, Cytology

## Abstract

**Background:**

A cross sectional study to investigate HPV prevalence according to age and cytology.

**Methods:**

Women presenting to a gynaecological outpatient clinic for a Pap smear test were included in the study (n=3177). All women had cervical cytology and HPV testing.

**Results:**

Overall prevalence of any 24 HPV type analysed was 33.1% (95% CI 31.5% to 34.7%) and HPV 16 and HPV 42 were the most frequent (6.7% (95% CI 5.8% to 7.6%), 6.8% (95% CI 5.9% to 7.6%)), in total samples. Multiple HPV infection rate was 12.9% (95% CI 11.8% to 14.1%). High risk HPV (hrHPV) types were present in 27.4% (95% CI 25.8% to 28.9%) of the samples.

HPV prevalence was highest among 14 to 19 y.o (46.6% (95% CI 40.7%-52.4%)) and second highest among 30–34 y.o. (39.7%, 95% CI 35.4%–44%). HPV 16 was highest among 20–24 (9.0% (95% CI 6.4%–11.6%)) and second highest among 50 to 54 y.o. (6.3% (95% CI 2.9% to 9.8%).

In Low-grade Squamous Intraepithelial Lesions (LgSIL) cytology samples, the most frequently detected hrHPV types were: 16 (14.5% (95% CI 12.1% to 16.9%)), 51 (13.0% (95% CI 10.7% to 15.3%)) and 53 (9.1% (95% CI 7.2% to 11.1%)) and in High-grade Squamous Intraepithelial Lesions (HgSIL) were: HPV 16 (37.2% (95% CI 26.5% to 47.9%)), HPV 51 (17.9% (95% CI 9.4% to 26.5%)) and HPV 18 (12.8% (95% CI 5.4% to 20.2%)).

**Conclusions:**

In the population studied, HPV 16 and 51 were the most frequent detected hrHPV types. HPV positivity, hrHPV and multiple HPV types infections were higher in young women, while HPV prevalence declined with increasing age and presented two peaks a higher (14–19 y.o.) and a lower one (30–34 y.o.) These results may contribute to the creation of a national screening programme.

## Background

Overall and age specific distribution of high risk HPV (hrHPV) and low risk HPV (lrHPV) types data across geographical regions is crucial for the optimization of prevention strategies in each country [1-4]. Over 100 types of HPV can infect the anogenital epithelium, but only 18 types, designated as “oncogenic”, can progress to severe lesions [5]. Most infections are encountered by the immune system and regress spontaneously without even been clinically detected. However, in certain individuals infections persist and if left untreated can lead to cervical intraepithelial lesions and cervical cancer.

HPV 16 and HPV 18 are the most common oncogenic types associated with cancer, and are targeted by recently developed vaccines [6-8]. The “non-oncogenic” HPV types are associated with hyperplastic lesions such as genital warts. Infection with a hrHPV is a major contributing factor in cervical cancer. HPV infection usually peaks in younger women indicating the age of their first sexual intercourse while the proportion of HgSIL cases is higher in middle aged women.

In order to improve the current cervical screening process, it is essential to combine cytology and HPV testing. Large scale randomized controlled studies need to be performed to establish the most efficient and cost effective cervical cancer screening programme. In Greece there are limited epidemiological data on HPV prevalence [9-12].

The aim of this study is to describe the prevalence and age distribution of different HPV types among women presenting for a Pap smear in an outpatient clinic in Greece.

## Methods

The study group consisted of a consecutive sample of 3177 Caucasian women between 14 and 70 years old who proceeded to the Outpatient Gynaecological Clinic of Regional Anticancer Oncology Hospital of Athens “St. Savvas” (participation rate 67%) and 1^st^ Department of Obstetrics and Gynaecology, University of Athens, ‘Alexandra’ Hospital (participation rate 33%), between January 2007 and November 2010 in order to have their annual test Pap.

The patients were also offered HPV test with the knowledge that it is not part of the screening. Women were eligible if they were no pregnant and had no history of HPV cervical disease. All patients included in the study have been only checked once throughout these years. Adequate specimens had been obtained from all of them, except 7, which were excluded. All patients included in the study gave their written informed consent after discussing with gyneacologists that there would be no implications to their health. Sexually active adolescents attended either an adolescent health clinic or an adolescent gynecology clinic in order to have gyneacological tests, such as conventional genital cultures, and were offered cervical screening and HPV typing as part of a sexual health prevention protocol. All adolescents and their guardians gave their written informed consent prior to being examined after discussing with gynaecologists that it would have no impact on their well being. Ethical approval was granted by the ethics committee of Regional Anticancer Oncology Hospital of Athens “St. Savvas”.

The studied population was divided into three age groups according to the biological alterations in women’s reproductive system (14–25 y.o., 26–46 y.o. and 47–70 y.o), in order to facilitate the recognition of specific differences in the biological and clinical behaviour of HPV infection across the years. Cervical cell scrapings were collected by a gynaecologist with a cytobrush (Rovers Cervex -Brush Combi Rovers Medical Devices B.V, The Netherlands) from the ecto- and endo-cervix of the uterus. A slide was prepared for conventional cytology and the cytobrush was then placed in specimen transport medium (Thin-Prep PreservCyt Solution Corporate Headquarters: Hologic, Inc.Ltd.UK) and stored at 4°C until prepared for HPV molecular analysis. Cytological findings were classified in line with the 2001 Bethesda classification system. The smears were submitted to two cytopathologists who worked separately and the results were discussed. In rare cases of discrepancy the smear was submitted to the Head of the Cytopathology department. The Cytopathology department has established an internal and also external quality control based on international external quality assurance tests (Labquality-Alpha Medical) 5 ml of Thin Prep samples were used for DNA isolation. They were centrifuged, diluted into lysis buffer (NucliSENS lysis buffer, bioMérieux Hellas S.A) and finally subjected to the NucliSENS® easyMAG®platform (bioMérieux Hellas S.A) for automated extraction, according to manufacturer’s instructions. Nucleic acids were eluted in 55 μl of elution buffer. DNA quality test was carried out using Human Globin, Beta, Primer set kit (Maxim Biotech, Inc., South San Francisco, CA) according to manufacturer’s instructions. Aliquots were stored appropriately for further processing.

The PapilloCheck® HPV-Screening (Greiner Bio-One GmbH, Germany) was used. This technology is based on a DNA chip for the type-specific identification of 24 HPV types (high risk: 16, 18, 31, 33, 35, 39, 45, 51, 52, 56, 58, 59, 68, 73, 82 probable high risk 53 and 66 and low risk: 6, 11, 40, 42, 43, 44/55, 70) [5]. Nucleic acids were extracted from the cervical scrape preserved in the ThinPrep. E1 based PCR was performed according manufacturer’s guidelines. For each sample we mixed 19.8 μl PapilloCheck® MasterMix, 0.2 μl HotStarTaq plus DNA polymerase (5U/μl, Qiagen®) and 5μl DNA from the cervical sample. Hybridization followed by mixing 30μl of the PapilloCheck® Hybridization buffer in a fresh reaction tube with 5 μl of the PCR-product at room temperature and transferring 25 μl of the hybridization mix into each compartment of the chip. Positive and negative controls were used. The chip was incubated for 15 minutes at 25°C temperature in a humid atmosphere and then washed in 3 solutions, centrifuged and scanned on the CheckScanner™.

Data were analysed using SAS v9.0. Absolute and relative frequencies were used to present the HPV types distribution according to age and cytology. The HPV prevalence and 95% confidence intervals were calculated using binomial methods and stratified by cytology report and HPV category. Chi-squared tests were performed to assess statistical significance of any differences in prevalence. 2 × 2 contingency tables Fisher’s exact test was performed along with Odds Ratio and 95% confidence intervals calculation. Pearson’s Chi Square test was used in cases of contingency tables bigger than 2 × 2. Cochran-Armitage test for trend was used to investigate trend in distribution of certain types and multiple HPV infection according to escalation of age. 2-independent samples t-test was used, along with relevant descriptive statistics (mean value, standard deviation and 95% confidence interval for mean value) to compare average age among patients depending on presence of specific types and multiple infections. 5% level of statistical significance and 95% confidence intervals were used.

## Results

The response rate was 62% (3177/5124) and the final sample consisted of 3170 women with complete cytological and HPV-DNA testing results. The majority of them were Greek (2993/3170, 94.4%) and a 5.6% (177/3170) were Caucasian immigrants living in Greece. Women were 14–70 years old (mean age 34.2) and were divided according to their age in three groups: 14-25 (858 women, range 11, mean 21.2, median 22), 26–46 y.o. (1760 women, range 20, mean 34.6, median 34) and 47-70 y.o (552 women, range 13, mean 53.0 , median 52). Their cytology results and distribution by age group are shown in Table 1.

**Table 1:** HPV (HPV+), High Risk HPV (HR+) and multi infection in cytology and age subgroups

		**Normal**		**ASCUS**		**LSIL**		**HSIL**		**All**	
		**N**	**% (95% CI)**	**N**	**% (95% CI)**	**N**	**% (95% CI)**	**N**	**% (95% CI)**	**N**	**% (95% CI)**
	Total	537	62.6 (59.3–65.8)	10	1.2 (0.4–1.9)	298	34.7 (31.5–37.9)	13	1.5 (0.7–2.3)	**858**	**27.1 (25.5–28.6)**
14–25	HPV+	129	24.0 (20.4–27.6)	7	70 (–)	221	74.2 (69.2–79.1)	13	100 (–)	**370**	**43.1 (39.8–46.4)**
	Multiple	39	7.3 (5.1–9.5)	2	20 (0–44.8)	115	38.6 (33.1–44.1)	10	76.9 (–)	**171**	**19.9 (17.3–22.6)**
	HR	107	19.9 (16.5–23.3)	7	70 (–)	199	66.8 (61.4–72.1)	13	100 (–)	**326**	**38 (34.7–41.2)**
	Total	1226	69.7 (67.5–71.8)	26	1.5 (0.9–2.0)	450	25.6 (23.5–27.6)	58	3.3 (2.5–4.1)	**1760**	**55.5 (53.8–57.3)**
26–46	HPV+	165	13.5 (11.5–15.4)	9	34.6 (16.3–52.9)	342	76 (72.1–79.9)	54	93.1 (–)	**570**	**32.4 (30.2–34.2)**
	Multiple	39	3.2 (2.2–4.2)	3	11.5 (0–23.8)	148	32.9 (28.5–37.2)	21	36.2 (23.8–48.6)	**211**	**12.0 (10.5–13.5)**
	HR	111	9.1 (7.4–10.2)	9	34.6 (16.3–52.9)	286	63.6 (59.1–68.0)	49	84.5 (75.2–93.8)	**455**	**25.9 (23.8–27.9)**
	Total	455	82.4 (79.3–85.6)	17	3.1 (1.6–4.5)	73	13.2 (10.4–16.1)	7	1.3 (0.3–2.2)	**552**	**17.4 (16.1–18.7)**
47–70	HPV+	54	11.9 (8.9–14.8)	2	11.8 (0–27.1)	48	65.8 (54.9–76.6)	5	71.4 (–)	**109**	**19.7 (16.4–23.1)**
	Multiple	17	3.7 (2.0–5.5)	0	0	14	19.2 (10.1–28.2)	0	0	**31**	**5.6 (3.7–7.5)**
	HR	41	9.0 (6.4–11.6)	2	11.8 (0–27.1)	39	53.4 (42.0–64.9)	5	71.4 (–)	**87**	**15.8 (12.7–18.8)**
	Total	**2218**	**70 (68.4–71.6)**	**53**	**1.7 (1.2–2.1)**	**821**	**25.9 (24.4–27.4)**	**78**	**2.5 (1.9–3)**	**3170**	**100 (2.5–3.8)**
**All**	HPV+	**348**	**15.7 (14.2–17.2)**	**18**	**34 (21.2–46.7)**	**611**	**74.4 (71.4–77.4)**	**72**	**92.3 (86.4–98.2)**	**1049**	**33.1 (31.5–34.7)**
	Multiple	**95**	**4.3 (3.4–5.1)**	**5**	**9.4 (1.6–17.3)**	**279**	**34 (30.7–37.2)**	**30**	**38.5 (27.7–49.3)**	**410**	**12.9 (11.8–14.1)**
	HR	**259**	**11.7 (10.3–13.0)**	**18**	**34 (21.2–46.7)**	**524**	**63.8 (60.5–67.1)**	**67**	**85.9 (78.2–93.6)**	**868**	**27.4 (25.8–28.9)**

**Figure 1:**
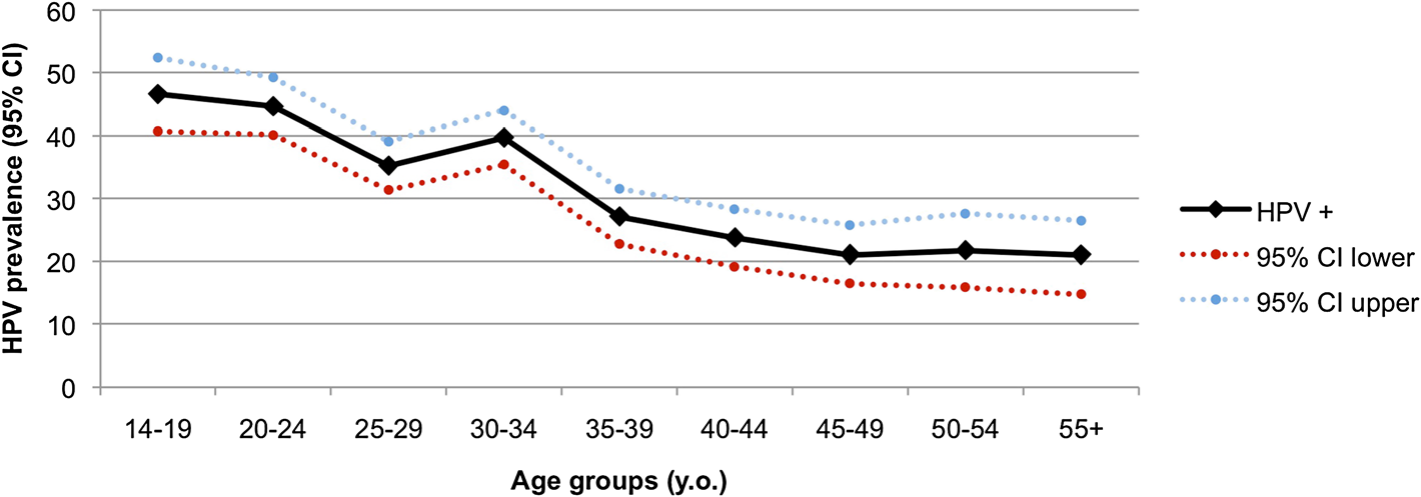
HPV prevalence (HPV+) in 5-year age groups.

Overall, 1049 of the 3170 (33.1% (95% CI 31.5% to 34.7%)) samples were found to harbour an HPV infection. We examined HPV prevalence in 5-year periods in order to assess age trends in relation to HPV infection in more detail. HPV prevalence exhibited a peak of 46.6% (95% CI 40.7%–52.4%) at 14–19 y.o. and a second peak at the age group 30-34 y.o. (39.7%, 95% CI 35.4%–44%) which decreased thereafter (Figure 1). It was also found that the presence of any HPV genotype was significantly more frequently identified in younger rather than older women (mean age of HPV positive women 31.2 vs 35.7 for HPV negative, t test p<0.001).

The overall HPV prevalence in normal, ASCUS, LSIL and HSIL were 15.7% (95% CI 14.2% to 17.2%, 348/2218), 34% (95% CI 21.2% to 46.7%, 18/53), 74.4% (95% CI 71.4% to 77.4%, 611/821) and 92.3% (95% CI 86.4% to 98.2%, 72/78), respectively (Table 1). As expected, HPV positivity was greater in higher grade cytology abnormalities either in overall or in each age group separately (Pearson’s Chi Square value p<0.001, Cochran-Armitage trend test p value p<0.001). Microarrays detected high risk HPV infection (HR+) in 868 of 3170 women (27.4%, 95% CI 25.8% to 28.9%). As far as cytology was concerned, the proportion of samples containing at least one of the 17 high risk types was greater in SIL (LSIL, HSIL 591/899, 65.7% (95% CI 62.6% to 68.8%)) than in ASCUS (18/53, 34% (95% CI 21.2% to 46.7%) Fisher’s exact test p value p<0.001) or normal cytology samples (259/2218, 11.7% (95% CI 10.3% to 13%) Fisher’s exact test p value p<0.001).

### HPV type distribution

Using the Microarrays method, 24 HPV types were detected (15 high risk types, 2 probable high risk and 7 low risk). Overall, HPV 16 was the most common high risk HPV type present with a prevalence of 6.7% (95% CI 5.8% to 7.6%), followed by HPV 51 (5.7%, 95% CI 4.9% to 6.6%), HPV 53 (3.8%, 95% CI 3.2% to 4.5%), HPV 56 (3.4%, 95% CI 2.7% to 4.0%) and HPV 31 (2.5%, 95% CI 2.0% to 3.1%) (Additional file 1: Table S1).

**Figure 2:**
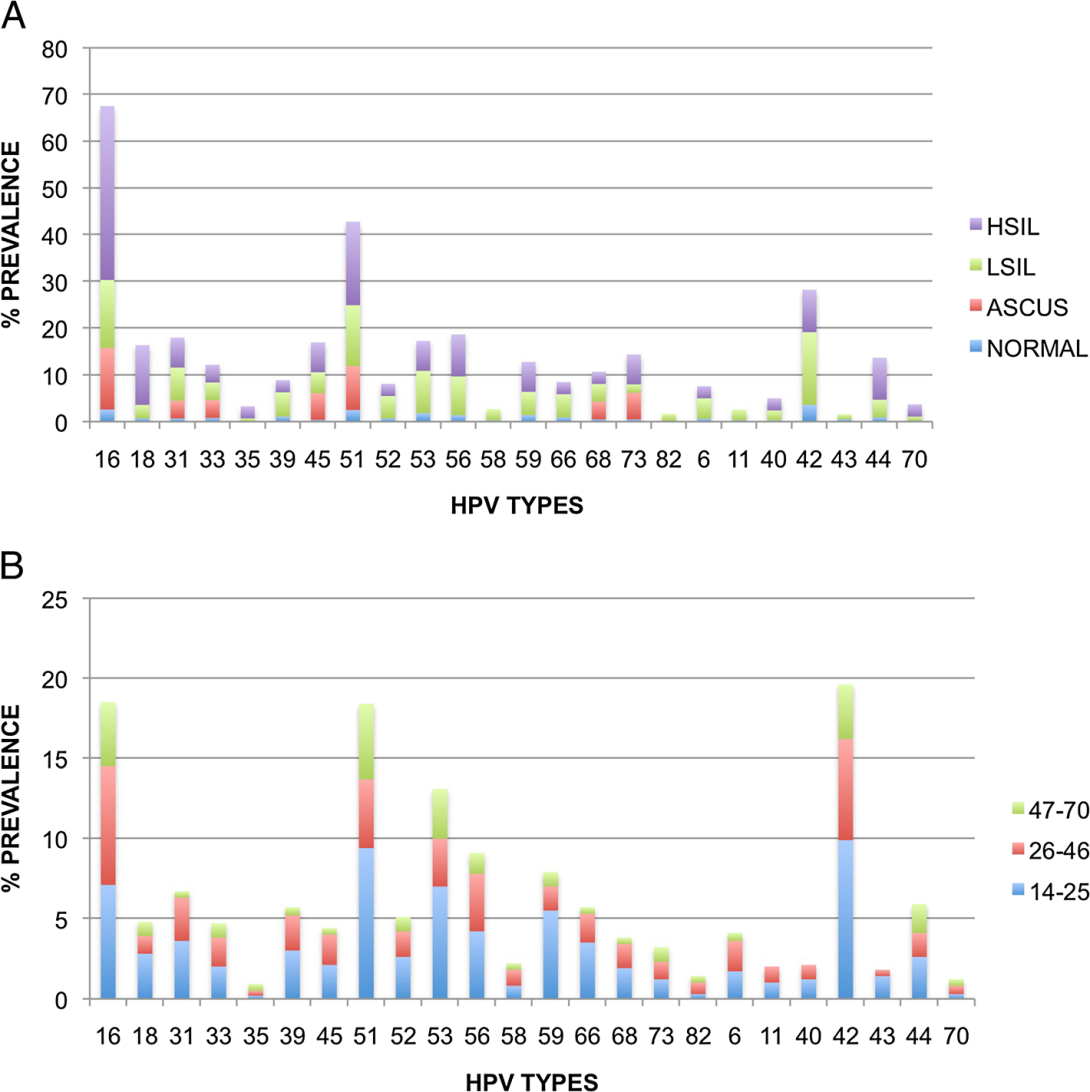
**The relationship between HPV types distribution and cytology (A) and age (B) (with full details given in Additional files**1**and**2**).**

Within the normal cytology samples, the most common high risk HPV types identified were 16 (2.6%, 95% CI 2.0% to 3.3%), 51 (2.5%, 95% CI 1.9% to 3.2%) and 53 (1.8%, 95% CI 1.3% to 2.4%). Among ASCUS cytology samples, only hrHPV types were detected; HPV 16 was once more the most frequent type (13.2%, 95% CI 4.1% to 22.3%) followed by HPV 51 (9.4%, 95% CI 1.6% to 17.3%) and HPV 45 (5.7%, 95% CI 0 to 11.9%). The three most frequently detected hr HPV types were 16 (14.5%, 95% CI 12.1% to 16.9%), 51 (13%, 95% CI 10.7% to 15.3%) and 53 (9.1%, 95% CI 7.2% to 11.1%) in LSIL. In HSIL the most common hrHPV types were 16 (37.2%, 95% CI 26.5% to 47.9%), 51 (17.9%, 95% CI 9.4% to 26.5%) and 18 (12.8%, 95% CI 5.4% to 20.2%) (Figure 2a).

Regarding the lrHPV types, HPV 42 was the most common HPV type with an overall prevalence of 6.8% (214, 95% CI 5.9% to 7.6%), followed by HPV 44 (1.9%, 95% CI 1.4% to 2.3%) and HPV 6 (1.6%, 95% CI 1.2% to 2.0%). These three lrHPV types were detected in this order in women with normal cytology (3.6% (95% CI 2.8% to 4.4%), 0.9% (95% CI 5.0% to 13.0%) and 0.6% (95% CI 3.0% to 10.0%) respectively). HPV 42 was the most frequent lrHPV type detected in LSIL (15.6%, 95% CI 13.1% to 18.1%) followed by HPV 6 (4.4%, 95% CI 3.0% to 5.8%) and HPV 44 (3.8%, 95% CI 2.5% to 5.1%) (Figure 2a).

The age-specific prevalences of HPV types are shown in Figure 2b. HPV 51 exhibited the highest peak (9.4%, 95% CI 7.5%–11.4%) among the hrHPV types at the age group 14–25. The prevalence of HPV 51 decreased thereafter, until the age 26-46. Similarly the peak of HPV 18 prevalence (2.8%, 95% CI 1.7%–3.9%) appeared in the age group 14–25, and decreased thereafter. Regarding lrHPV types, HPV 42 was prevalent throughout all ages, presenting a peak of 9.9% (95% CI 7.9%–11.9%) at the age group 14–25 and decreasing thereafter until 3.4% (95% CI 1.9%–5.0%) in women 47–40. The prevalence peak of HPV 6 (1.9%, 95% CI 1.2%–2.5%) was detected in the 26–46 years age group, and then dropped until 0.5% (95% CI 0–1.2%) in the 47–70 years age group. HPV 11 presented equally low percentages in the two younger groups and did not appear at all in the oldest women. (Additional file 2: Table S2).

**Figure 3:**
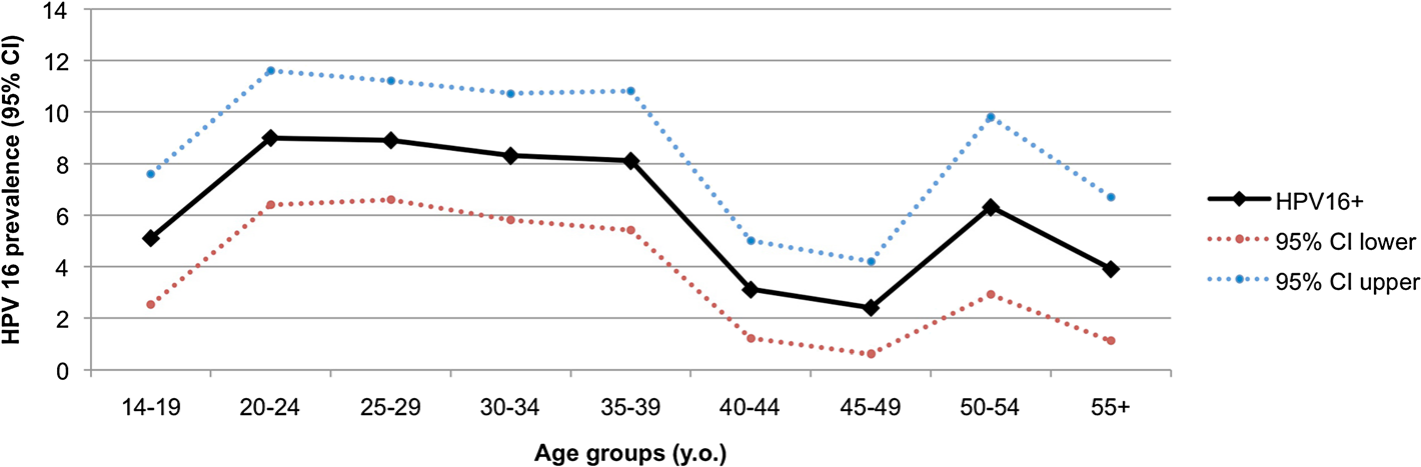
Prevalence curve of hrHPV type 16 in 5-year age groups.

HPV 16 was the most prevalent HPV type in total and in order to acquire more information regarding its age distribution we studied it in 5-year age groups. The first peak appeared at the age group 20–24 (9%, 95% CI 6.4%–11.6%) and HPV 16 prevalence remained relatively high until the age of 35-39 y.o. and decreased sharply thereafter. A second peak of 6.3% (95% CI 2.9%–9.8%) was present at women aged 50–54 y.o and the prevalence dropped in older women (Figure 3).

### Multiple HPV infection

Multiple HPV infection with either lr or hr HPV types was found in 12.9% (410/3170, 95% CI 11.8% to 14.1%) of all samples and 39.1% (410/1049, 95% CI 36.1% to 42%) of the HPV infected samples (Table 1). The prevalence of multiple HPV infection was higher in the 14–25 age group and reduced in older ages. It was also found that the mean age of multiple HPV infected women (30.1) was lower than that of single HPV positive women (32.0), a difference that was statistically significant (t test p=0.036).

Multiple HPV positivity was significantly more common in SIL (LSIL, HSIL, 309/899, 34.4% (95% CI 31.3% to 37.5%)) than in ASCUS (5/53, 9.4% (95% CI 1.6% to 17.3%), Fisher’s exact test p value p<0.001) or normal (95/2218, 4.3% (95% CI 3.4% to 5.1%), Fisher’s exact test p value p<0.001).

Among women with multiple infections, 66.8% (95% CI 62.3% to 71.4%) were infected with two HPV types; the most common combination was of 16 with 51, 56 with 66 and 6 with 42. There were 22.6% (95% CI 18.6% to 26.7%) of infected women with three HPV types, the most common combinations of which were 11 with 33 and 45 and the combination of 16 with 42 and 45. There were also cases with 4 HPV types (8.3%, 95% CI 5.6% to 11.0%), 5 HPV types (1.4%, 95% CI 0.3% to 2.6%) and 6 HPV types (1%, 95% CI 0.0% to 1.9%).

## Discussion

We present a large study of HPV prevalence in women of different ages in the area of Athens, from which interesting conclusions can be drawn. As HPV DNA typing is gradually incorporated in national screening programmes knowledge of HPV type variations in different geographical regions is useful information. Similarly, as HPV vaccination is introduced as a means of primary cervical cancer prevention, better prediction models of its efficacy specific for each area can be made [13].

In our study and in concordance with what is seen in the general population, normal cytology dominated all age groups. The highest LgSIL rates appeared among women aged 14–25 years. Only a minority (1.5%) of young girls had a high grade lesion and this is again consistent with what is seen in the literature. It also justifies the current American and British screening programme that suggest smear testing after the age of 21 and 25 respectively [14,15].

In our study, the HPV prevalence in normal samples was 15.7%. For Eastern Europe, HPV prevalence in a meta-analysis of 4053 samples tested with normal cytology was 21.4% [4]. Studies from different regions of Greece have reported an overall HPV prevalence ranging from 22.7 to 50.7% [11,12] which is in accordance to our HPV prevalence of 33.1%. Our findings are also in agreement with other studies in Greece that have suggested a prevalence of hrHPV ranging between one in five and one in three. It does however come in stark difference with what has been reported in another Greek study conducted by the University of Thessaloniki [10], where a prevalence of only 2.5% was reported which is among the lowest ever reported in the world. This difference might be attributed to the fact that this latter study used a different HPV identification method.

In an attempt to compare our results with other countries’ epidemiological data, we presented an age-related prevalence curve of HPV infection. This curve showed a peak at the 14–19 age group, a second one at 30–34 y.o. and then dropped. Although the study by Coupe et al conducted in the Netherlands demonstrated an association between age and HPV prevalence [16], others have not identified a significant relation [17]. When we looked specifically at the age related prevalence of hrHPV, we observed a peak in the 14–25 years age group, in accordance with other European studies [18,19]. Younger women are more prone to develop an HPV infection as they tend to have multiple partners [20] and are also less likely to have developed immunity to HPV given their recent exposure to the virus.

As expected, HPV prevalence, increased as lesions progressed to higher grade ones. The same trend was observed in hrHPV positivity in relation to cytological status. Investigating the results per age group, we observed that hrHPV infection dominated in 14-25 year old women irrespective of cytology. However young women are less likely to present cancerous lesions as in the majority of cases the lesion regresses after 2–3 years [21,22].

Previously, in a 1636 women cohort study conducted by this group, where the prevalence of subtypes 6, 11, 16, 18, 31 and 33 was tested, HPV 11 was found to be the most frequent [9]. In the current study, where a wider range of HPV subtypes were investigated, HPV 16 and 42 were the most frequent (6.7% and 6.8% respectively) in total samples followed by HPV 51 (5.7%). HPV 51 was constantly identified as a common HPV type in all age groups however its ranking dropped significantly behind HPV 16 in ages 26–46. A possible explanation for this may be that HPV 51, although highly prevalent, is cleared more quickly and possibly causes a more consistent immunologic response with a longer immune protection conferred to those that have cleared the virus, making them no longer susceptible to new infections.

HrHPV distribution in Mediterranean countries like Italy, Portugal, Spain [23-26] is similar to our data with HPV 16 detected first and HPV 51 ranking second or third. Although HPV 18 prevalence was overall low, its presence was high (12.8%) among women with high grade lesions. Generally, the predominance of HPV 16 and 18 in high grade lesions strengthens the importance of vaccination in prevention of cervical cancer as 50% of the HSIL lesions in our study harboured infection by these two types.

Multiple infections were found in 12.9% of Greek population. Multiple HPV positivity was more common in younger women. In addition, women with cervical lesions had a higher rate of multiple infections compared to those who had normal cytology. Multiple infections might be a risk factor for development of cytological abnormalities. The majority of multi-HPV infected women harboured at least one hrHPV type and the majority of those carried two types, which is consistent with other epidemiological studies [1].

Although this study presented here included a large number of women of a broad age range, it has some limitations. Our sample is representative of women presenting for smear testing. However, as smear taking in Greece is done on an ad hoc basis rather through an organised screening system, our sample cannot be considered representative of the Athenian population. These data may give us important information regarding regional HPV prevalence but large epidemiological studies from different regions of our country are needed.

## Conclusions

HPV 16 and 51 were the most commonly identified hrHPV types, present in 6.7% and 5.7% overall and 37.2% and 17.9% in HgSIL cytology samples, respectively. Multi infection was present in 39.1% of infected cytology samples. Our results were comparable with what has been found in other Mediterranean countries.

HPV positivity, hrHPV and multiple HPV types infections were higher in young women. HPV prevalence declined with increasing age and presented two peaks a higher (14–19 y.o.) and a lower one (30–34 y.o.). HPV 16 prevalence remained high until 35-39 y.o. and presented a second peak in women aged 50–54 y.o. These results may contribute to the creation of a national screening programme.

## Competing interests

The authors declare that there are no competing interests.

## Authors’ contributions

EA carried out the molecular tests, participated in the statistical analysis and drafted the manuscript. SP and ET carried out the molecular tests, participated in the statistical analysis and helped to draft the manuscript. LM and EM performed gynaecologic examinations and helped to draft the manuscript. IP participated in the statistical analysis and helped to draft the manuscript. DD performed the cytologic analysis. IT and GM performed gynaecologic examinations. EP designed and coordinated the study and helped to draft the manuscript. All authors read and approved the final manuscript.

## Pre-publication history

The pre-publication history for this paper can be accessed here:

http://www.biomedcentral.com/1471-2334/13/53/prepub

## Supplementary Material

Additional file 1 HPV type distribution according to the cytology subgroups. Click here for file

Additional file 2 HPV type distribution according to the age groups. Click here for file
